# ApoE4 antibody ameliorates cognitive impairments and synapse loss in ApoE4/Tau‐P301S mice

**DOI:** 10.1002/alz.088537

**Published:** 2025-01-09

**Authors:** Ha‐Lim Song, Min‐Seok Kim, Mi‐Hyang Cho, Yeon‐Seon Mun, Na‐Young Kim, Jung‐Su Shin, Jun‐Sub Kim, Dong‐Hou Kim, Seung‐Yong Yoon

**Affiliations:** ^1^ ADEL Institute of Science & Technology (AIST), ADEL, Inc., Seoul Korea, Republic of (South); ^2^ Asan Medical Center, University of Ulsan College of Medicine, Seoul Korea, Republic of (South)

## Abstract

**Background:**

The Apolipoprotein E4 isoform (ApoE4), encoded by the APOE gene, stands out as the most influential genetic factor in late‐onset Alzheimer’s disease (LOAD). The ApoE4 isoform contributes to metabolic and neuropathological abnormalities during brain aging, with a strong correlation observed in APOE4‐positive Alzheimer’s disease cases between phosphorylated tau burden and amyloid deposition. Despite compelling evidence of APOE‐mediated neuroinflammation influencing the progression of tau‐mediated neurodegeneration, the molecular mechanisms underlying these phenomena remain largely unknown.

**Method:**

Utilizing Tau‐P301S mice crossed to APOE4‐knockin mice expressing human APOE4 (TauP301S/APOE4), the investigation tested the impact of anti‐mouse ApoE4 antibody, ADEL‐Y04, on memory enhancement and tau pathology reduction.

**Result:**

We developed ADEL‐Y04, an anti‐mouse ApoE4 antibody specifically recognizing human ApoE4. Administration of ADEL‐Y04 in TauP301S/APOE4 mice demonstrated improvements in memory impairment. Synapse loss and pathological tau accumulation was also inhibited by ADEL‐Y04 administration.

**Conclusion:**

Our findings suggest the potential for improving APOE4‐mediated tau pathology and memory impairment through the modulation of ApoE4. This provides avenues for intervention, indicating the possibility of ameliorating tau pathology and memory deficits by targeting APOE4.

